# Pamrevlumab, a Fully Human Monoclonal Antibody Targeting Connective Tissue Growth Factor, for Non-Ambulatory Patients with Duchenne Muscular Dystrophy

**DOI:** 10.3233/JND-230019

**Published:** 2023-07-04

**Authors:** Anne M. Connolly, Craig M. Zaidman, John F. Brandsema, Han C. Phan, Cuixia Tian, Xueping Zhang, Jack Li, Mark D. Eisner, Ewa Carrier

**Affiliations:** a Nationwide Children’s Hospital, Ohio State University College of Medicine, Columbus, OH, USA; b Department of Neurology, Washington University at St. Louis, St. Louis, MO, USA; c Division of Neurology, The Children’s Hospital of Philadelphia, Perelman School of Medicine, University of Pennsylvania, Philadelphia, PA, USA; d Rare Disease Research, LLC, Atlanta, GA, USA; e Division of Neurology, Cincinnati Children’s Hospital Medical Center, Cincinnati, OH, USA; f Department of Pediatrics, University of Cincinnati College of Medicine, Cincinnati, OH, USA; g FibroGen, Inc., San Francisco, CA, USA

**Keywords:** Clinical trial, connective tissue growth factor, Duchenne muscular dystrophy, percent predicted forced vital capacity, grip strength, monoclonal antibody, pamrevlumab

## Abstract

**BACKGROUND:**

Duchenne muscular dystrophy (DMD) is a neuromuscular disease stemming from dystrophin gene mutations. Lack of dystrophin leads to progressive muscle damage and replacement of muscle with fibrotic and adipose tissue. Pamrevlumab (FG-3019), a fully human monoclonal antibody that binds to connective tissue growth factor (CTGF), is in Phase III development for treatment of DMD and other diseases.

**METHODS:**

MISSION (Study 079; NCT02606136) was an open-label, Phase II, single-arm trial of pamrevlumab in 21 non-ambulatory patients with DMD (aged≥12 years, receiving corticosteroids) who received 35-mg/kg intravenous infusions every 2 weeks for 2 years. The primary endpoint was change from baseline in percent predicted forced vital capacity (ppFVC). Secondary endpoints included other pulmonary function tests, upper limb function and strength assessments, and changes in upper arm fat and fibrosis scores on magnetic resonance imaging.

**RESULTS:**

Fifteen patients completed the trial. Annual change from baseline (SE) in ppFVC was –4.2 (0.7) (95% CI –5.5, –2.8). Rate of decline in ppFVC in pamrevlumab-treated patients was slower than observed in historical published trials of non-ambulatory patients. MISSION participants experienced slower-than-anticipated muscle function declines compared with natural history and historical published trials of non-ambulatory patients with DMD. Pamrevlumab was well-tolerated. Treatment-emergent adverse events were mild to moderate, and none led to study discontinuation.

**CONCLUSIONS:**

nti-CTGF therapy with pamrevlumab represents a potential treatment for DMD. The lack of internal control group limits the results.

## INTRODUCTION

Duchenne muscular dystrophy (DMD), the most common inherited neuromuscular disease of childhood, arises from a genetic mutation in the dystrophin gene (locus Xp21.2) [1–4]. Males are primarily affected [2]. X-linked recessive inheritance is common, and the disorder can also arise from spontaneous mutations [2]. *DMD* gene mutations cause a decrease in or an absence of dystrophin protein, an essential structural component of muscle tissue, leading to progressive skeletal, respiratory, and cardiac muscle degeneration, as well as replacement with fibrotic and adipose tissue [2]. Progressive skeletal muscle damage and fibrosis lead to loss of ambulation at around 12 years of age. As arm weakness progresses, patients become increasingly dependent on others for daily activities [1–4]. Degeneration and weakness of respiratory and cardiac muscles lead to restrictive pulmonary disease and heart failure, which are the leading causes of morbidity and mortality in patients with DMD [2].

Corticosteroids are considered the standard of care in DMD to improve strength and pulmonary function [5]. With the use of corticosteroids, a delay in pulmonary function decline by 2–3 years has been observed. However, once patients are in the decline phase, a similar rate of decline has been observed, regardless of corticosteroid treatment [6–8]. In addition to corticosteroids, several therapies that target specific *DMD* gene mutations amenable to exon skipping (eteplirsen, golodirsen, viltolarsen, casimersen) have been granted accelerated approval by the U.S. Food and Drug Administration (FDA). While each has provided small increases in dystrophin expression, clinical benefits have been variable and frequently modest [9–17].

**Fig. 1:**
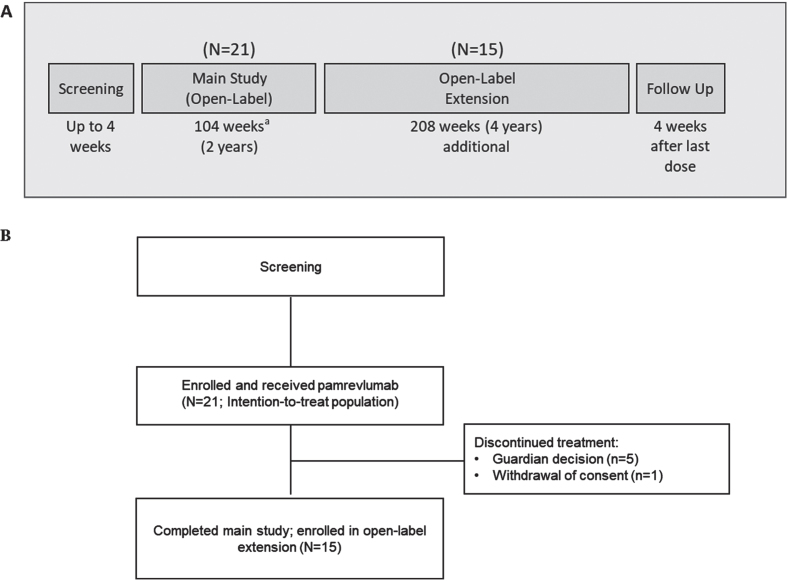
(A) Study design (B) Patient disposition. ^a^Two patients were in the main study for 206 weeks.

Fibrosis in DMD has been linked to overexpression of connective tissue growth factor (CTGF), a secreted extracellular matrix glycoprotein produced by various cell types including fibroblasts, myofibroblasts, and endothelial cells [18, 19]. CTGF interacts with a variety of regulatory modulators, such as transforming growth factor-*β*, vascular endothelial growth factor, and integrin receptors, modulating normal processes involved in tissue repair and pathologic processes involved in fibrosis. Skeletal muscle from both patients with DMD and dystrophic dogs exhibited elevated concentrations of CTGF [20, 21], and overexpression of CTGF induced muscle damage and decreased muscle strength in wild-type mice similar to the damage observed in *mdx* mice (used as a murine model for DMD) [18]. Cardiac dysfunction and fibrosis are also major manifestations of DMD. In the *mdx* mouse heart, this fibrosis was associated with increased CTGF expression [18]. CTGF may be a key mediator of early and persistent fibrosis in dystrophic cardiomyopathy [22].

Pamrevlumab (FG-3019), a fully human monoclonal antibody targeting CTGF, has led to reductions in fibrosis and improvements in function in skeletal and cardiac muscle in preclinical models of DMD. In a study of *mdx* mice, inhibition of CTGF (either through administration of an anti-CTGF monoclonal antibody or through gene therapy) inhibited muscle fibrosis and improved muscle strength and exercise capacity [23]. Anti-CTGF monoclonal antibody treatment also reduced progression of sensorimotor decline and fibrosis in a rat model of chronic repetitive muscle overuse [24] and inhibited skeletal muscle fibrosis after denervation in mice [25]. Anti-CTGF monoclonal antibody inhibition of CTGF in an Emery-Dreifuss mouse model of dilated cardiomyopathy attenuated cardiac fibrosis and improved skeletal muscle function [26]. A chimeric antibody similar to pamrevlumab has also demonstrated some effects on fibrosis markers and tissue remodeling in pressure overload–induced heart failure [27], myocardial infarction [28], and another genetically engineered model of dilated cardiomyopathy [29]. Together, these observations suggest that CTGF plays an important role in DMD and that inhibition of CTGF by pamrevlumab could decrease fibrosis and improve skeletal and cardiac muscle function.

The primary objective of MISSION was to examine the efficacy of pamrevlumab in non-ambulatory patients with DMD. Secondary objectives included safety, tolerability, and pharmacokinetic (PK) assessments.

## MATERIALS AND METHODS

### Study design and oversight

MISSION was an open-label, Phase II, single-arm study of pamrevlumab in non-ambulatory patients with DMD conducted by 10 investigators at 10 sites in the United States. The study consisted of a 4-week screening period, a 104-week main study period, a 208-week open-label extension period, and a follow-up period (Fig. 1A). Results of the main study period are reported here. The study was conducted and monitored in accordance with FDA regulations, the International Council for Harmonisation E6 Guideline for Good Clinical Practice, the Declaration of Helsinki, and any other applicable regulatory requirements. The research protocol was approved by a relevant institutional review board, and all participants provided written informed consent or assent.

### Patients

Included in this study were non-ambulatory patients≥12 years with a diagnosis of DMD and a confirmed *DMD* gene mutation identified through genetic testing. Patients had a Brooke Upper Extremity scale score of≤5, a percent predicted forced vital capacity (ppFVC) between 40% and 90%, and a left ventricular ejection fraction (LVEF)≥45% on cardiac magnetic resonance imaging (MRI). Patients had to have been receiving stable dosages of corticosteroids for≥6 months prior to screening, with no change in dosage for≥3 months other than adjustments for body weight. Those receiving medications for heart failure had to have achieved a stable regimen for≥3 months prior to screening. Excluded were patients requiring≥16 hours per day of continuous ventilation, those with a prior or ongoing medical condition that could have impacted the safety of the patient and/or the ability to fulfill study obligations, and those with a hospitalization due to respiratory failure in the prior 6 weeks. Participants could not have received another investigational or approved drug for DMD in the 28 days before the start of study treatment, with the exception of corticosteroids. Complete inclusion and exclusion criteria are available in Supplementary Appendix S1.

### Study medication/assessments

Following the 4-week screening period, all participants received pamrevlumab at a dosage of 35 mg/kg intravenous every 2 weeks. The first infusion was based on the body weight obtained during screening. Dosage was adjusted based on body weight and was assessed approximately every 3 months thereafter. Patients whose weight exceeded 117 kg during the course of the study received the maximum allowed dose of 4.1 g. The dosage was determined based on results of a study of adults with pancreatic cancer and was projected to achieve a minimum C_max_ of 150μg/mL. The dosing interval was based on safety and efficacy findings from clinical experience with pamrevlumab.

Vital signs and adverse events were monitored at each 2-week visit. Weight and height (estimated from ulnar length) were measured at screening and every 3 months thereafter. Physical examination, pulmonary function tests, and muscle function tests were conducted at screening, on Day 0, every 12 weeks thereafter through Week 84, and at Week 104. Laboratory assessments were conducted at baseline, at Week 4, at Week 8, and then on the same schedule as function tests and physical exam. Muscle MRI, cardiac MRI, and electrocardiograms were obtained at baseline and at Weeks 52 and 104. Approximately 30% of patients were unable to complete a Week-104 ppFVC assessment, only 6 patients completed a Week-104 biceps brachii MRI, and only four patients completed a Week-104 cardiac MRI. (Of note, lockdowns and delays because of SARS-CoV-2 [COVID-19] in the United States began in March 2020, approximately 8 weeks before the last patient completed the study. Specifically, COVID-19 restrictions were noted as the causes of nine missed appointments or assessments.)

Spirometric pulmonary function tests included ppFVC, percent predicted forced expiratory volume in 1 second (ppFEV_1_), and percent predicted peak expiratory flow rate (ppPEF). Muscle function tests included the Performance of Upper Limb (PUL 2.0) score, and grip strength and pinch strength obtained via hand-held myometry. T2 MRI mapping of the upper arm (biceps brachii) was used to determine a muscle fat and fibrosis score. Cardiac MRI measures included fibrosis score and LVEF. Cardiac fibrosis and other cardiac outcomes will be published separately.

Blood samples for PK assessments were collected at pre-dose, within 1 hour after end of the infusion of pamrevlumab, and on Days 2, 4, 7, 10, and 14 following the first dose. Steady-state samples were obtained at Weeks 26 and 52 (pre-dose at both time points and post-dose at Week 52). Pamrevlumab concentrations were measured in all samples. PK parameters were calculated from the concentration versus time data from each patient by standard noncompartmental methods (Phoenix64^®^, WinNonlin^®^, Build 8.1, Certara, Princeton, NJ).

### Study endpoints/statistical analysis

All efficacy endpoints were based on the intention-to-treat population (all patients who enrolled in the study). The primary endpoint was the annual rate of change from baseline to Week 104 in ppFVC during treatment with pamrevlumab. FVC was selected because it was deemed the best assessment involving all respiratory muscles, requiring both a full inspiration (reflecting function of inspiratory muscles) and a full expiration (reflecting function of expiratory muscles) [30]. It is a reliable, responsive, and clinically meaningful measure of DMD progression [30]. Secondary pulmonary function endpoints were the changes from baseline to Week 104 in ppFEV_1_ and ppPEF.

Muscle function endpoints included mean change from baseline to Week 104 in PUL 2.0 total score, middle arm score, and distal arm score. The recently developed PUL Version 2.0 was used, which eliminates some redundancies and simplifies scoring compared with the previous version (i.e., Version 1.2), while maintaining its reliability and improving its ability to capture change across the range of DMD severities [31–33].

Also analyzed were the change from baseline to Week 104 in grip strength and pinch strength by hand-held myometry, fat fraction percentage (% F) by MRI, and biceps brachii muscle fat and fibrosis score by T2 MRI mapping. T2 mapping is a biomarker that can help determine the degree of fibrosis, inflammation, edema, and fat infiltration present in the affected muscle [34, 35]. Differences in T2 relaxation time of normal versus pathologic (e.g., fibrotic or fatty) tissue types may be used to diagnose disease, measure the severity of involvement, and monitor therapeutic response. Exploratory endpoints included the PK profile and laboratory measures. A *post-hoc* analysis of change from baseline in Brooke Upper Extremity Scale score from baseline to Week 104 was also performed. An additional *post-hoc* analysis was performed on changes in grip strength in patients with baseline Brooke scores of≤4 versus patients with baseline scores of 5.

This study evaluated whether pamrevlumab could attenuate the annual decline from baseline to Week 104 in ppFVC in non-ambulatory patients with DMD. A total of 22 participants were planned to achieve 80% power to test the null hypothesis of change in ppFVC of –5%, the same change noted in historical published data [36]. This null hypothesis was tested against the alternative hypothesis, assuming a mean change of –2% and standard deviation of 5% based on two-sided one-sample *t*-test at 0.05 significance level.

The primary endpoint of annual change in ppFVC (i.e., the mean of changes occurring between Years 1 and 2) was analyzed using a random coefficient model. This model included visit in years as a continuous variable, baseline ppFVC as a fixed effect, and the intercept and visit as random effects. The same analysis model was used in all other functional endpoints. For patients with at least one post-baseline FVC assessment, observed data at all post-baseline visits were included in the model. Missing data were not imputed. For the other endpoints (i.e., upper arm fibrosis and fat score, and % F), the same random coefficient model was used. Exploratory subgroup analyses assessed whether the type of corticosteroid (i.e., prednisone or deflazacort) or patient age (i.e.,≤16 or > 16 years) affected the change from baseline in pulmonary or muscle function endpoints.

A subset (N = 36) of matched patients from the Cooperative International Neuromuscular Research Group (CINRG) DMD Natural History Study (DNHS) [8] was included in the analyses as an external group to compare changes in FVC and grip strength. The CINRG DNHS is the largest prospective multicenter natural history study in DMD, encompassing≥10 years of follow up in ≥400 patients. The 36 non-ambulatory patients were selected for comparison based on age, corticosteroid use, and baseline function assessments (comparison against historical control data is a pragmatic strategy in rare disease trials) [37]. Corticosteroid dosages and schedules were not available for the CINRG cohort: data were only available to indicate if a patient was or was not using corticosteroids at the time of study entry, and this was the basis for the match with the patients of the MISSION cohort. Data for all compared endpoints were available for all 36 patients. In addition, various prospective published data were used as historical comparisons. These studies were selected based on non-ambulatory patient status, similarity of endpoints to the MISSION study, and availability of 1- or 2-year results [32, 36, 38–40]. Specifically, the Phase III DELOS trial was chosen as the comparator for pulmonary function. This study included a well-defined cohort of patients with DMD aged 10–18 years who were not receiving corticosteroids [38]. While this population is not a direct match with our corticosteroid-treated patients, the authors believe it is a reasonable and justifiable comparison since it provides an expanded understanding of the natural course of pulmonary disease in DMD. In addition, once patients with DMD begin to decline (as expected in the teenage boys included in this study and in the historical comparator), the rate of pulmonary decline in DMD is the same for those treated or not treated with corticosteroids [6–8, 30].

Descriptive summaries for change from baseline by analysis visit, annual rate of change from baseline (analyzed using a random coefficient model), and the estimated change from baseline values at Years 1 or 2 (i.e., Weeks 52 or 104) for the comparisons to external data were implemented for the primary and secondary efficacy endpoints. The most comparable published historical control data for the updated PUL 2.0 instrument [32, 33] were not prespecified in the Statistical Analysis Plan and are considered *post hoc*.

### Role of the funding source

The trial was designed by staff of FibroGen, Inc. Data were collected by local site investigators and were analyzed and interpreted by FibroGen in collaboration with the authors. All authors had full access to the trial data following final database lock and provided critical review and input. The corresponding author had final responsibility for the decision to submit for publication.

## RESULTS

### Patient disposition/baseline characteristics

Twenty-one patients were enrolled in the main study and received at least one dose of pamrevlumab (Fig. 1B). The first patient was enrolled on January 4, 2016, and the last patient completed the main study on May 7, 2020. Fifteen patients completed the main study and were enrolled in the open-label extension. Five patients withdrew during the main study period because of guardian decisions, and one additional patient withdrew consent after the last study visit at Week 104. (Two patients, both≤16 years of age, were enrolled in the main study for 206 weeks. All assessments were included in the random coefficient model analysis. Inclusion of the two patients’ data from visits beyond 2 years did not significantly impact the results.) All patients were included in the intention-to-treat and safety populations.

Demographics and baseline DMD disease history are provided in Table 1, and baseline assessments are listed in Table 2. All 21 patients were male,≥12 years of age, and non-ambulatory, with a genetically confirmed DMD diagnosis (specific mutation categories are provided in Supplementary Appendix S2). All patients were receiving corticosteroids (43% deflazacort and 57% prednisone), with the majority on a daily regimen. Corticosteroid treatment was started at a median age of 6 years, corresponding to a mean (SD) length of therapy of 8.7 (3.4) years (range 1.1, 16.6 years). The most common conditions cited in the medical history were femur fracture (33.3%), restrictive lung disease (28.6%), headache/migraine (28.6%), scoliosis (23.8%), tenotomy (19%), asthenia (19%), and sleep apnea (19%).

**Table 1:** Demographics and baseline DMD disease history

	MISSION	CINRG DNHS^8^	*p*-value
	(N = 21)	(N = 36)
Age, y
Mean (SD)	16.0 (3.3)	14.6 (2.0)	*p* = 0.043
Median (range)	15.8 (12.4, 25.6)	14.2 (12.0, 19.4)
≤16, *n* (%)	12 (57.1)
17–18, *n* (%)	6 (28.6)
>18, *n* (%)	3 (14.3)
Male sex, *n* (%)	21 (100.0)	36 (100.0)
Race, *n* (%)
White	20 (95.2)	29 (80.6%)	*p* = 0.56
Black or African American		1 (2.8%)
Asian	1 (4.8)	3 (8.3%)
Other	3 (8.3%)
Weight, kg
Mean (SD)	64.9 (20.1)	48.6 (16.0)	*p* = 0.023
Median (range)	63.5 (28.3, 110.6)	43.4 (29.0, 90.0)
BMI, kg/m^2^
Mean (SD)	24.9 (7.2)	21.4 (5.3)	*p* = 0.058
Median (range)	24.8 (12.2, 36.1)	20.8 (13.4, 34.9)
Height, cm
Mean (SD)	161.4 (7.9)	149.8 (12.8)	*p* = 0.0010
Median (range)	159.1 (149, 177)	146.2 (132.0, 178.2)
BSA, m^2^
Mean (SD)	1.7 (0.3)	1.4 (0.3)	*p* = 0.0007
Median (range)	1.7 (1.1, 2.2)	1.3 (1.1, 2.0)
Dominant arm, *n* (%)		—	—
Left	1 (4.8)
Right	20 (95.2)
Age at diagnosis, y		—	—
Mean (SD)	5.5 (3.1)
Median (range)	5.5 (0.6, 12.2)
Age when patient became non-ambulatory, y		—	—
Mean (SD)	11.9 (1.8)
Median (range)	12.0 (9, 15)
Years since patient became non-ambulatory		—	—
Mean (SD)	4.1 (2.7)
Median (range)	3.4 (1, 11.5)
Genetic characteristics, *n* (%)		—	—
Exon deletion	12 (57.1)
Duplication	4 (19.0)
Point mutation	3 (14.3)
None of the above	2 (9.5)
Corticosteroid use, *n* (%)			—
Deflazacort	9 (42.9)	29 (80.6)
Prednisone	12 (57.1)	7 (19.4)
Daily use	16 (76.2)	—
Twice weekly use	5 (23.8)	—
Age when patient began corticosteroids, y		—	—
Mean (SD)	7.3 (3.6)
Median (range)	6.0 (3.0, 17.0)

Abbreviations: BMI = body mass index; BSA = body surface area; CINRG = Cooperative International Neuromuscular Research Group; DMD = Duchenne muscular dystrophy; DNHS = DMD Natural History Study; SD = standard deviation.

**Table 2:** Baseline assessments

	MISSION	CINRG DNHS^8^
	(N = 21)	(N = 36)
ppFVC (%)
Mean (SE)	54.2 (2.5)	66.8 (12.2)
Median (range)	54.2 (29.1, 70.7)	66.5 (44.0, 88.0)
ppPEF (%)		—
Mean (SE)	54.7 (2.7)
Median (range)	52.4 (37.9, 82.7)
ppFEV_1_ (%)		—
Mean (SE)	53.8 (2.7)
Median (range)	55.2 (29.2, 73.4)
Upper limb (PUL) score, total		—
Mean (SE)	24.4 (2.0)
Median (range)	22 (13, 41)
Upper limb (PUL) score, middle arm		—
Mean (SE)	10.1 (1.0)
Median (range)	10 (4, 17)
Upper limb (PUL) score, distal arm		—
Mean (SE)	11.0 (0.2)
Median (range)	11 (8, 13)
Brooke upper extremity scale score
Mean (SD)	3.3 (1.5)	2.7 (1.2)
Median (range)	3.0 (1.0, 5.0)	3.0 (1.0, 5.0)
Grip strength, dominant hand, newtons
Mean (SE)	45.9 (7.9)	58.6 (26.0)
Median (range)	37.0 (3, 142)	53.2 (13, 121.5)
Grip strength, non-dominant hand, newtons		—
Mean (SE)	42.0 (6.7)
Median (range)	37.0 (2, 104.9)
Pinch strength, dominant hand, newtons		—
Mean (SE)	17.0 (2.9)
Median (range)	14.0 (0, 45.1)
CAD assessment of muscle fat and fibrosis (mean T2 mapping within the bicep ROI) (1/s)		—
	*n* = 12
Mean (SE)	8.0 (1.0)
Median (range)	7.5 (3.9, 17.2)
Fat fraction (%)	*n* = 9	—
Mean (SE)	22.1 (3.0)
Median (range)	24.2 (4, 32.6)

Abbreviations: CAD = computer-aided detection; CINRG = Cooperative International Neuromuscular Research Group; DMD = Duchenne muscular dystrophy; DNHS = DMD Natural History Study; ppFEV_1_ = percent predicted forced expiratory volume in 1 second; ppFVC = percent predicted forced vital capacity; ppPEF = percent predicted peak expiratory flow rate; PUL = performance of the upper limb; ROI = region of interest; SD = standard deviation; SE = standard error.

Baseline measures from the patients in the CINRG database [8] are also provided in Table 1 for comparison. At the time of entry into the CINRG study, all patients were taking corticosteroids (81% deflazacort and 19% prednisone), with a mean (SD) length of therapy of 7.2 (2.7) years (range 3.0, 14.1 years). There was no significant difference between the MISSION cohort and the CINRG patients in the duration of corticosteroid use before or during the study. The pamrevlumab group was significantly older and taller, with significantly greater weight and body surface area. Study designs and relevant baseline assessments for the historical comparisons are provided in Supplementary Appendix S3 [32, 36, 38–40].

### Pulmonary function assessments

The annual change from baseline (SE) in ppFVC with pamrevlumab, the primary endpoint, was –4.2 per year (0.7; 95% CI –5.5, –2.8), with similar declines observed during Year 1 (least-squares estimate of the mean change from baseline –4.0 [0.9; 95% CI –5.8, –2.2]) and Year 2 (least-squares estimate of the mean change from baseline –8.2 [1.1; 95% CI –10.3, –6.0]) (Table 3) [36, 38].

**Table 3:** Mean change from baseline on functional outcomes for MISSION vs. historical controls^32,36,38–40^

Assessment					
	ppFVC	PUL (v2.0)	PUL (v2.0)	PUL (v2.0)	Grip strength	Grip strength
		total score	middle arm score	distal arm score	(dominant hand),	(non-dominant
					newtons	hand) newtons
**MISSION ( N** = **21)**
Annual change (95% CI)	–4.2 (–5.5, –2.8)	–2.2 (–3.1, –1.2)	–0.9 (–1.5, –0.4)	–0.2 (–0.4, –0.1)	N/A^b^	N/A^b^
1 year (95% CI)	*n* = 19	*n* = 19	*n* = 19	*n* = 19	*n* = 19	*n* = 19
	–4.0 (–5.8, –2.2)	–2.0 (–2.9, –1.1)	–0.7 (–1.3, –0.1)	–0.1 (–0.4, 0.2)	1.0 (–5.9, 8.0)	1.9 (–4.9, 8.6)
2 years (95% CI)	*n* = 15	*n* = 18	*n* = 18	*n* = 18	*n* = 18	*n* = 18
	–8.2 (–10.3, –6.0)	–4.1 (–5.4, –2.9)	–1.6 (–2.5, –0.77)	–0.3 (–0.7, 0.2)	–2.5 (–9.6, 4.6)	–1.3 (–8.4, 5.8)
**CINRG DNHS ( N** = **36)**
1 year (95% CI)	–6.9 (–9.6, –4.2)				–1.9 (–4.9, 1.1)
*p*-value^a^	*p* = 0.078				*p* = 0.450
2 years (95% CI)	–10.7 (–13.4, –8.1)				–5.0 (–8.0, –2.1)
*p*-value^a^	*p* = 0.140				*p* = 0.525
**Ricotti 2019 ( N** = **29)**
1 year (95% CI)	–5.5 (–6.5, –4.5)				–3.8 (–4.9, –2.8)
*p*-value^a^	*p* = 0.170				*p* = 0.188
**Meier 2017 ( N** = **33)**
1 year (all placebo; N = 33) (95% CI)	–8.7 (–11.0, –6.5)
*p*-value^a^	*p* = 0.0018
1 year (prior GC use; *n* = 19) (95% CI)	–8.7 (–11.4, –5.9)
*p*-value^a^	*p* = 0.0057
^c^**Mayhew 2020 ( N** = **90)**
1 year (95% CI)		–2.2 (–2.9, –1.4)	–1.2 (–1.6, –0.7)	–0.4 (–0.6, –0.1)
*p*-value^a^		*p* = 0.74	*p* = 0.18	*p* = 0.12
2 years (95% CI)		–4.4 (–5.3, –3.4)	–2.4 (–2.9, –1.9)	–0.8 (–1.0, –0.5)
*p*-value^a^		*p* = 0.71	*p* = 0.15	*p* = 0.078
**Seferian 2015 ( N** = **53)**
1 year (95% CI)					–2.7 (–4.9, –0.6)	–3.0 (–4.6, –1.5)
*p*-value^a^					*p* = 0.32	*p* = 0.174

^a^All *p*-values are versus MISSION change from baseline. ^b^Change in grip strength was not linearly distributed over time, so estimates of annual change are unreliable. ^c^For Mayhew, the PUL total score analysis was *post-hoc*, as were all statistical comparisons vs. MISSION. Abbreviations: CI = confidence interval; CINRG = Cooperative International Neuromuscular Research Group; DMD = Duchenne muscular dystrophy; DNHS = DMD Natural History Study; GC = glucocorticoid; N/A = not applicable; ppFVC = percent predicted forced vital capacity; PUL = performance of upper limb.

The 1-year decline in ppFVC was less than the declines observed in prospective published trials of non-ambulatory patients encompassing 1-year follow up [36, 38]. The difference at 1 year was statistically significant in favor of pamrevlumab (–4.0 [–5.8, –2.2]) versus the total placebo group (–8.7 [–11.0, –6.5] [*p* = 0.0018]) and a subset of that group (i.e., prior glucocorticoid therapy) (–8.7 [–11.4, –5.9] [*p* = 0.0057]) of the Phase III DELOS study [38]. No significant difference at 1 year or 2 years was observed compared with the CINRG natural history study group (Table 3) [36, 38]. Results of pulmonary function secondary endpoints (i.e., ppFEV_1_ and ppPEF) through Week 104 are listed in Supplementary Appendix S4 [36, 38]. There was little evidence of an effect for patient age or corticosteroid use on lung function (Supplementary Appendix S5).

### Left ventricular ejection fraction

The least-squares estimate of the mean change (SE) from baseline in LVEF% was –0.02 (1.29; 95% CI –2.9, 2.9) at 1 year and –2.7 (1.7; 95% CI –6.4, 1.0) at 2 years. At Year 1, the LVEF% decline was smaller for pamrevlumab than for historical published data for corticosteroid users (–0.02 vs. –0.8) [8]. Historical data were not available for a 2-year comparison.

### Upper limb function assessment

The annual change from baseline (SE) in PUL total score with pamrevlumab was –2.2 (0.48; 95% CI –3.1, –1.2). The least-squares estimate of the mean change from baseline was –2.00 (0.45; 95% CI –2.9, –1.1) at Year 1 and –4.1 (0.65; 95% CI –5.4, –2.9) at Year 2 (Table 3) [32, 39]. For the middle and distal arm scores, the annual changes were –0.9 (95% CI –1.5, –0.4) and –0.2 (95% CI –0.4, 0.1), respectively.

PUL outcomes from MISSION were compared with outcomes from a prospective 2-year study by Mayhew A, et al. (Table 3) [32, 39]. The mean baseline PUL total score was approximately 5 points lower than the baseline score in MISSION (19.7 vs. 24.4). Despite this, the magnitude of decline was similar at Years 1 and 2.

**Fig. 2:**
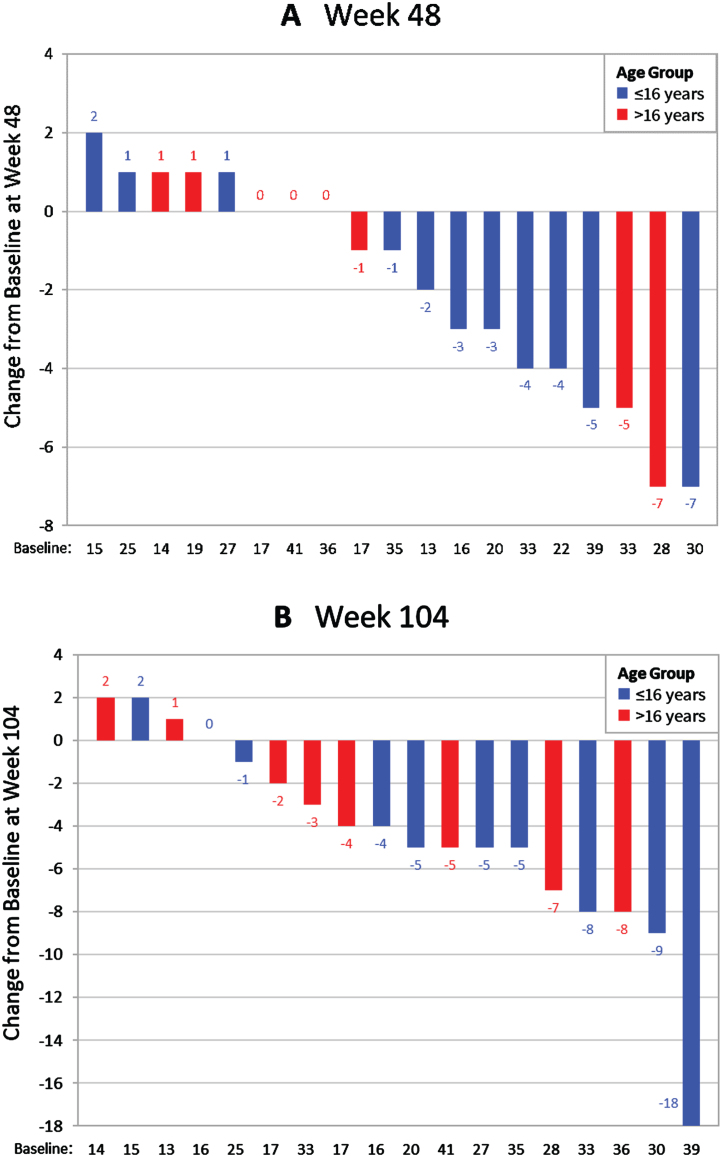
Waterfall plots showing the distribution of change from baseline in PUL 2.0 total scores at (A) Week 48 (1 year) (*n* = 19) and (B) Week 104 (2 years) (*n* = 18).

There were no significant differences between MISSION and the 2-year prospective comparison on any PUL measure. However, PUL scores varied between patients. A total of 42.1% (8/19) of patients did not experience a decline in PUL score at 1 year, and 27.8% (4/18) did not experience a decline at 2 years. The percentages not experiencing a decline in distal arm score were 68.4% (13/19) and 66.7% (12/18), respectively. Several patients experienced improvement or stability in PUL scores at both time points (Fig. 2).

A *post-hoc* analysis assessed changes in function in MISSION as measured on the Brooke Upper Extremity Scale. The 1-year mean change from baseline (0.23 [0.099]) and 2-year mean change from baseline (0.4 [0.1]) both demonstrated slight score increases (scale is 1 to 6, with greater scores representing lower function).

### Myometric strength assessments

Grip strength in MISSION increased slightly in Year 1 and then decreased in Year 2. The least-squares estimate of the mean change from baseline was 1.0 (3.51; 95% CI –5.9, 8.0) at Year 1 and –2.5 (3.61; 95% CI –9.6, 4.6) at Year 2. Similar patterns occurred in grip strength in the non-dominant hand. Pinch strength scores are reported in Supplementary Appendix S5 [36, 40].

Some patients attained improvements in dominant hand grip strength up to the first year of pamrevlumab treatment, irrespective of age (Supplementary Appendix S6). After that, there was a moderate decline in grip strength for patients older than 16 years, versus some stabilization in younger patients. Grip strength performance was generally better, but more variable, with prednisone than with deflazacort.

In a *post-hoc* analysis, gains in grip strength through Year 1 were observed in those with Brooke scores≤4 at baseline (2.7 [5.6]), but not in those with Brooke scores of 5 (–1.4 [1.4]). Thus, grip strength improvements were achieved in patients who were stronger at baseline.

These results are similar to those for patients in the CINRG DNHS and published historical data. The studies used for comparison saw decreases in grip strength in the first year (Table 3) in either the dominant or non-dominant hand, although none of the differences were significant compared with the present study [36, 40].

At baseline, the CINRG participants had a mean (SE) grip strength in the dominant hand of 58.6 (26.0) newtons, which was greater than the 45.9 newtons in the MISSION participants. Consequently, grip strength remained greater for the CINRG group throughout the entire 2-year period (Supplementary Appendix S7).

### Skeletal muscle assessments

Nine patients underwent % F assessments with MRI at baseline and at Years 1 and 2. From a mean (SE) baseline of 22.1% (3.0), fat increased on average by 3.3% /year (95% CI –2.1, 8.6), with most increases occurring during Year 2.

Twelve patients underwent T2 mapping within the biceps brachii region of interest at baseline and Years 1 and 2. The mean (SE) T2 mapping score at baseline was 8.0 (1.0). The least-squares estimate of the mean change from baseline was –2.6 (95% CI –4.3, –0.9) at 1 year and –2.22 (95% CI –4.6, 0.1) at 2 years. A positive correlation was observed between the change in biceps brachii T2 mapping and change in PUL total score at 1 year (Spearman correlation = 0.7, *p* = 0.029) and 2 years (Spearman correlation = 0.5, *p* = 0.288).

### Pharmacokinetics

Twelve patients were included in the PK analysis. The concentration profiles were similar for patients aged > 16 years compared with those aged≤16 years. The maximum concentration was reached 2.7 hours after the start of the pamrevlumab infusion. Clearance and apparent volume of distribution at steady state were 0.2 mL/h/kg and 52 mL/kg, respectively, with a mean terminal half-life of 9.2 days (Supplementary Appendix S8). There was no difference between minimum concentration at Week 26 compared with Week 52 (mean [SD], 655.5 [186.5] vs 738.8 [161.9] μg/mL, respectively), which suggests that patients reached steady state by Week 26.

### Safety

The most common treatment-emergent adverse events (TEAEs) reported in≥25% of patients were flu-like symptoms, including headache (66.7%), nasopharyngitis (52.4%), vomiting (47.6%), cough (42.9%), pyrexia (38.1%), back pain (38.1%), nausea (33.3%), and sinus congestion (28.6%).

Table 4 is a summary of TEAEs occurring in≥2 patients. Although all patients experienced at least one TEAE during the treatment period, 61.8% of these events were Grade 1 (28.6%) or Grade 2 (33.3%). A total of 38.1% of patients experienced at least one severe (≥Grade 3) TEAE, but most of these were single occurrences in either one or multiple system organ classes. No TEAEs led to pamrevlumab or study discontinuation. Approximately half (47.6%) of patients experienced a TEAE that was considered related to the study medication. The majority were nervous system or gastrointestinal system related, with the most common being headache.

**Table 4:** Treatment-emergent adverse events occurring in≥2 patients

Preferred Term (MedDRA Version 18.1)	Pamrevlumab
	(N = 21) *n* (%)
Headache	14 (66.7)
Nasopharyngitis	11 (52.4)
Vomiting	10 (47.6)
Cough	9 (42.9)
Pyrexia	8 (38.1)
Back pain	8 (38.1)
Nausea	7 (33.3)
Sinus congestion	6 (28.6)
Abdominal pain upper	5 (23.8)
Diarrhea	5 (23.8)
Upper respiratory tract infection	5 (23.8)
Myalgia	5 (23.8)
Oropharyngeal pain	4 (19.0)
Rhinorrhea	4 (19.0)
Nasal congestion	3 (14.3)
Palpitations	3 (14.3)
Ear pain	3 (14.3)
Sinusitis	3 (14.3)
Dizziness	3 (14.3)
Anxiety	3 (14.3)
Cataract	2 (9.5)
Abdominal distension	2 (9.5)
Dyspepsia	2 (9.5)
Hypersensitivity	2 (9.5)
Influenza	2 (9.5)
Pneumonia	2 (9.5)
Muscle strain	2 (9.5)
Cystatin C increased	2 (9.5)
Weight decreased	2 (9.5)
Arthralgia	2 (9.5)
Migraine	2 (9.5)
Sinus headache	2 (9.5)
Depression	2 (9.5)
Nephrolithiasis	2 (9.5)
Productive cough	2 (9.5)
Erythema	2 (9.5)
Rash	2 (9.5)
Skin discoloration	2 (9.5)

Abbreviations: MedDRA = Medical Dictionary of Regulatory Activities.

One death occurred after withdrawal of consent and approximately 5 to 6 weeks after the last dose of pamrevlumab. Per the investigator, the death was deemed a result of disease progression and not related to pamrevlumab.

Six patients had treatment-emergent serious adverse events (SAEs), although none were deemed related to study drug by the investigators. The SAEs reported were a case of food poisoning leading to metabolic acidosis, a tramadol-related adverse drug reaction leading to hypotension, pneumonia, concussion and skull fracture secondary to trauma, femur fracture secondary to trauma, and nephrolithiasis with hydronephrosis. No clinically meaningful trends in laboratory measures were identified. No clinically important trends in electrocardiograms were observed.

## DISCUSSION

In this trial of non-ambulatory patients with DMD, the fully human monoclonal antibody pamrevlumab was associated with significantly less decline in ppFVC at 1 year than would be expected based on historical prospective data. The decline in ppFVC was numerically less than the CINRG cohort at 1 year and 2 years, but the confidence intervals were wide and overlapping. Pamrevlumab was well-tolerated in this population of non-ambulatory patients with DMD. The most common TEAEs, occurring in≥25% of patients, were flu-like symptoms and headache.

On average, the patients in this Phase II study (MISSION) continued to experience declines in functioning over 2 years. However, there was some variability in the results. The findings that > 40% of patients did not decline in PUL score at 1 year and that > 25% did not decline after 2 years are of note for a non-ambulatory population. It is possible that the findings may represent a floor effect of the PUL. However, the PUL 2.0 was designed specifically to address both floor and ceiling effects, and a direct comparison of data using PUL 1.2 and PUL 2.0 showed that the floor effect in the latter was negligible [32]. A small number of patients achieved changes in their PUL and grip strength scores at 1 year, but it is unclear whether these changes represent a true treatment effect of pamrevlumab or are simply a result of variability inherent in DMD. Placebo-controlled trials are needed to confirm efficacy. Two global, randomized, double-blind, placebo-controlled, Phase III trials of pamrevlumab in combination with systemic corticosteroids are well underway —one of non-ambulatory patients (LELANTOS-1; NCT04371666) and the other of ambulatory patients (LELANTOS-2; NCT04632940). These trials will evaluate the efficacy and safety of pamrevlumab for the treatment of DMD.

MISSION had several limitations that would prevent drawing definitive conclusions on efficacy. First, it was a small trial, with only 21 patients, and follow-up pulmonary function and cardiac testing were impacted by the COVID-19 pandemic. Second, this was an open-label, single-arm study. Finally, all comparisons described above are with unmatched historical cohort data. Although using historical comparisons is a common and accepted strategy in rare disease trials, results should be interpreted with caution because of differences in patient numbers, baseline characteristics, inclusion/exclusion criteria, treatment protocols, and analysis methods. The natural course of DMD is also variable, which complicates comparisons with external data.

CTGF inhibition with pamrevlumab is undergoing Phase III trials to evaluate the efficacy and safety for DMD, a genetic disease that continues to have unmet medical need. Cell, gene, and related therapies often provide inefficient delivery through muscle, and induced immunogenicity and potential off-target effects remain [41]. Therapies that target downstream mediators (e.g., CTGF and other targets [41]) may provide benefit in a broad range of patients, potentially without the genotype limitations and safety concerns of cell and gene therapies.

## Supplementary Material

Supplementary Material

## DATA SHARING

FibroGen, Inc., is committed to data sharing and to furthering medical research and patient care. Based on scientific merit, requests from qualified external researchers for anonymised patient-level and study-level clinical trial data (including redacted clinical study reports) for medicines and indications approved in the United States and Europe will be considered after the respective primary study is accepted for publication. All data provided are anonymised to respect the privacy of patients who have participated in the trial in line with applicable laws and regulations.
