# Supplementary material for: Pamrevlumab, a Fully Human Monoclonal Antibody Targeting Connective Tissue Growth Factor, for Non-Ambulatory Patients with Duchenne Muscular Dystrophy
Source: J Neuromuscul Dis. 2023 Jul 4;10(4):685–99. doi: 10.3233/JND-230019 (PMC10357134; doi:10.3233/JND-230019)
Supplement: Supplementary Material [file jnd-10-jnd230019-s001.pdf]

SUPPLEMENTARY APPENDIX

This appendix formed part of the original submission and has been peer reviewed.
We post it as supplied by the authors.

Supplement to: Connolly AM, Zaidman CM, Brandsema JF, et al. Pamrevlumab, a fully human monoclonal antibody targeting connective tissue growth factor, for non-ambulatory patients with Duchenne muscular dystrophy.

[full citation to come]

TABLE OF CONTENTS

S1 Inclusion and exclusion criteria ---48

S2 History at baseline visit and *DMD* gene mutation categories ---50

S3 Design and baseline descriptions for historical comparison studies---51

S4 Other pulmonary function endpoints ---52

S5 Subanalyses of mean (SE) change from baseline by visit ---53

S6 Mean change from baseline in pinch strength---54

S7 Mean (SE) change from baseline in ppFVC and grip strength in MISSION vs. CINRG-----------------------------55

S8 Preliminary PK parameters following a single dose of pamrevlumab (n=12) --56

Supplementary Appendix S1 Inclusion and exclusion criteria

Inclusion criteria

1. At least 12 years of age
2. Written consent/assent by patient and/or legal guardian as per regional and/or IRB requirements
3. Non-ambulatory
4. Brooke Score for Arms and Shoulders ≤5
5. Diagnosis of DMD by medical history and confirmed Duchenne mutation in available genetic testing using a validated genetic test
6. Able to perform spirometry
7. Able to undergo cardiac and extremity (upper arm) MRI
8. Percent predicted forced vital capacity (FVC) between 40 and 90, inclusive
9. At least one historical ppFVC value within 18 months of baseline
10. LVEF ≥45% as determined by cardiac MRI at screening or within 3 months prior to Day 0
11. Subjects currently receiving heart failure cardiac medications (e.g., angiotensin converting enzyme inhibitors, angiotensin-receptor blockers, and beta-blockers) had to achieve a stable regimen for at least 3 months prior to screening
12. Receiving a stable dosage of corticosteroids for a minimum of 6 months prior to screening with no substantial change in dosage for a minimum of 3 months (except for adjustments for changes in body weight) prior to screening and no foreseen change in corticosteroid use during the course of study
13. Received pneumococcal vaccine and was receiving annual influenza vaccinations
14. Adequate renal function: cystatin C ≤1.4 mg/L
15. Adequate hematologic function:
    a. Platelets >100,000/μL
    b. Hemoglobin >12 g/dL
    c. Absolute neutrophil count >1500/μL
16. Adequate hepatic function:
    a. No history or evidence of liver disease

   b. Gamma glutamyl transferase (GGT) ≤3 × upper limit of normal (ULN)
   c. Total bilirubin ≤1.5 × ULN
17. If sexually active, used medically accepted contraceptives during participation in the study and for 3 months after the last dose of study drug

Exclusion criteria

1. Required ≥16 hours continuous ventilation
2. Prior or ongoing medical condition that, in the investigator's opinion, could have adversely affected the safety of the subject, made it unlikely that the course of 156 weeks of treatment and follow up would be completed, or could have impaired the assessment of study results
3. Anticipated spine surgery within 156 weeks
4. Severe uncontrolled heart disease, including any of the following:
   a. Need for IV diuretics or inotropic support within 3 months prior to screening
   b. Hospitalization for a heart failure exacerbation or arrhythmia in the last 3 months
5. Arrhythmia requiring anti-arrhythmic therapy
6. Hospitalization due to respiratory failure in the last 6 weeks
7. Poorly controlled asthma or underlying lung disease, such as bronchopulmonary dysplasia
8. Known or suspected active hepatitis B or C or history of human immunodeficiency virus
9. Body mass index ≥40 kg/m^2 or weight >117 kg
10. Exposure to another investigational drug or another approved product for DMD (e.g., eteplirsen or golodirsen) within 28 days prior to start of study treatment (or 5 half-lives of the product, whichever is longer) prior to first Screening Visit, with the exception of deflazacort. Use of deflazacort, if regarded by the Principal Investigator as standard of care, was allowed.

Supplementary Appendix S2 History at baseline visit and *DMD* mutation categories

Patient/age	Years since patient became non-ambulatory	Years of corticosteroid use	Genetic characteristic
7901-1003/>16	5.3	5.3	Exon deletion
7903-3002/≤16	3.8	10.8	Exon deletion
7903-3003/≤16	1.8	9.8	Exon deletion
7903-3004/≤16	2.	8.7	None of the above
7903-3004/≤16	1.1	9.1	Duplication
7904-4003/≤16	3.4	6.4	Duplication
7904-4004/>16	10.6	16.6	Duplication
7904-4005/>16	4.4	9.4	Point mutation
7904-4006/>16	11.5	9.5	Exon deletion
7905-5004/≤16	2.4	7.2	Point mutation
7908-8003/≤16	3.8	8.8	Exon deletion
7908-8004/>16	1	7	None of the above
7908-8005/≤16	1.6	9.6	Point mutation
7908-8007/≤16	3.1	8.6	Exon deletion
7910-0103/>16	3.3	12.8	Exon deletion
7910-0104/≤16	3.9	9.9	Exon deletion
7912-1201/≤16	3.7	9.7	Duplication
7912-1203/>16	3.1	11.1	Exon deletion
7913-1002/≤16	3	1.1	Exon deletion
7920-0202/>16	6.2	10.2	Exon deletion
7921-2102/>16	7.1	1.1	Exon deletion

Supplementary Appendix S3 Design and baseline descriptions for historical comparison studies [1–5]

	Ricotti 2019 (*N*=29)	Meier 2017 (all-placebo group) (*N*=33)[a]	Pane 2018 Mayhew 2020 (*N*=90)	Seferian 2015 (*N*=53)
Trial design	Prospective natural history study	Prospective, randomized, placebo-controlled, Phase III trial (DELOS)	Prospective 2-year study	Observational, multicenter trial
Mean age, y (range)	14.2 (8.4, 18)	15	16.42 (9.08, 24.78)	17.1 (9.0, 28.1)
Male sex, *n* (%)	29 (100)	—	90 (100)	—
Non-ambulatory, *n* (%)	29 (100)	31 (93.9)	90 (100)	53 (100)
Corticosteroid use, *n* (%)	24 (82.7)	19 (57.6)	52 (57.8)	8 (15)
**Pulmonary function assessments**				
ppFVC, mean	62.10 (range 28, 108)	50.4 (SD 20.0)	—	44.5 (range 8, 104)
ppPEF, mean	65.6 (range 22.1, 107)	54.2 (SD 13.2)	—	—
$ppFEV_1$, mean	—	49.5 (SD 20.6)	—	—
**Upper limb strength assessments (PUL v2.0 score)**				
Total, mean (SD)	—	—	19.7 (10.9)	—
Middle arm, mean (SD)	—	—	7.77 (5.8)	—
Distal arm, mean (SD)	—	—	9.86 (2.6)	
**Grip and pinch strength assessment[b]**				
Grip strength, dominant hand, newtons, mean (range)	63.7 (5.2, 134.8)	—	—	—
Pinch strength, dominant hand, newtons, mean (range)	19.9 (2.7, 38.3)	—	—	—

[a]The DELOS trial included patients aged 10–18 years with a diagnosis of DMD who had stopped taking glucocorticoids at least 12 months prior to enrollment; they were not allowed to take glucocorticoids during the study period.

[b]Reported as kg and converted to newtons.

Abbreviations: PUL = performance of upper limb; ppFVC = percent predicted forced vital capacity; ppPEF = percent predicted peak expiratory flow rate; $ppFEV_1$ = percent predicted forced expiratory volume in 1 second; SD = standard deviation.

Supplementary Appendix S4 Other pulmonary function endpoints [2,3]

	MISSION (N=21)			Ricotti 2019 (N=29)		Meier 2017 (N=33)			
	Annual Change (95% CI)	1 year (95% CI)	2 years (95% CI)	1 year (95% CI)	*p*-value[a]	1 year All Placebo n = 33 (95% CI)	*p*-value[a]	1 year (Prior GC Use) n = 19 (95% CI)	*p*-value[a]
ppPEF,	−4.8 (−6.7, −2.9)	*n*=19 −2.4 (−6.6, 1.9)	*n*=14 −7.1 (−11.7, −2.6)	−4.8 (−6.8, −2.8)	*p*=0.32	−8.9 (−13.0, −4.7)	*p*=0.032	−7.8 (−13.5, −2.2)	*p*=0.126
$ppFEV_1$	−4.1 (−5.7, −2.5)	*n*=18 −4.2 (−6.7, −1.8)	*n*=15 −8.3 (−10.7, −5.9)	—	—	−10.2 (−14.2, −6.2)	*p*=0.0143	−8.7 (−12.7, −4.7)	*p*=0.0575

[a]All *p*-values are versus MISSION change from baseline.

Abbreviations: CI = confidence interval; GC = glucocorticoid; ppPEF = percent predicted peak expiratory flow rate; $ppFEV_1$ = percent predicted forced expiratory volume in 1 second.

Supplementary Appendix S5 Subanalyses of mean (SE) change from baseline by visit

Panels A–C subgroup analyses are based on patient age. Panels D–F are based on corticosteroid type.

Abbreviations: ppFVC = percent predicted forced vital capacity; PUL = performance of upper limb; SE = standard error.

A ppFVC

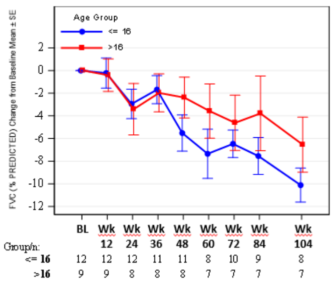

B PUL total score

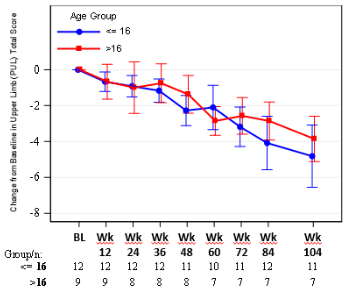

C Grip strength, dominant hand

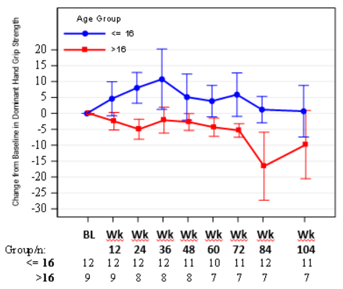

D ppFVC

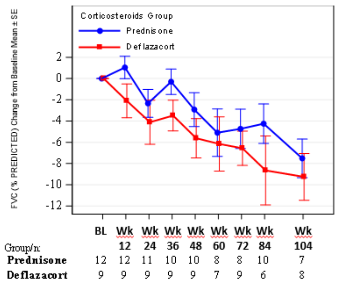

E PUL total score

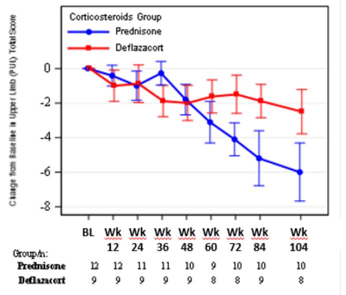

F Grip strength, dominant hand

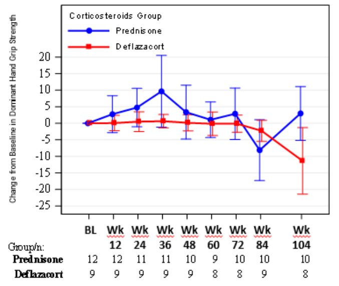

Supplementary Appendix S6 Mean change from baseline in pinch strength [2,5]

	**MISSION (N=21)**			**Ricotti (2019) (N=29)**		**Seferian (2015) (N=53)**	
	**Annual change (95% CI)**	**1 year (95% CI)**	**2 years (95% CI)**	**1 year (95% CI)**	***p*-value[a]**	**1 year (95% CI)[b]**	***p*-value[a]**
Pinch strength (dominant hand), newtons	N/A[b]	*n*=19 −1.8 (−3.5, 0.01)	*n*=16 −4.2 (−7.3, −1.2)	−0.8 (−1.3, −0.3)	*p*=0.30	−1.7 (−2.6, −0.8)	*p*=0.92
Pinch strength (non-dominant hand), newtons	N/A[b]	*n*=19 −1.96 (−3.8, −0.1)	*n*=16 −3.5 (−6.2, −0.7)	—	—	−2.2 (−3.2, −1.2)	*p*=0.85

[a]*p*-values are versus MISSION change from baseline.
[b]Change in grip strength was not linearly distributed over time, so estimates of annual change are unreliable.
Abbreviations: CI = confidence interval; N/A = not applicable.

Supplementary Appendix S7 Mean (SE) change from baseline in (A) ppFVC and (B) grip strength (dominant hand) by visit in MISSION and the CINRG DNHS

A ppFVC

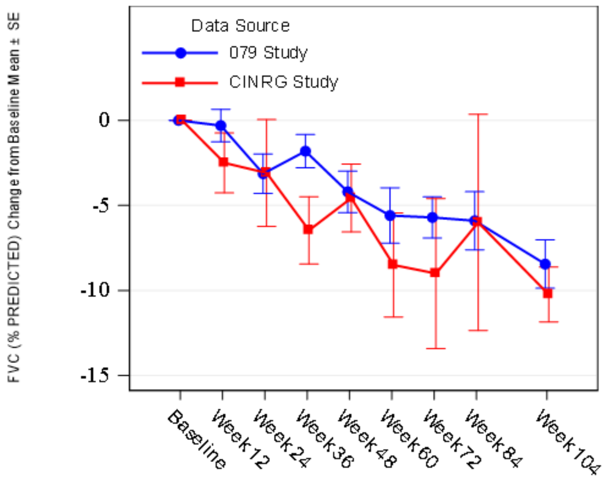

Group/n:									
**079 Study**	21	21	20	19	19	15	17	16	15
**CINRG Study**	36	10	11	13	19	12	7	6	19

B Grip strength, dominant hand

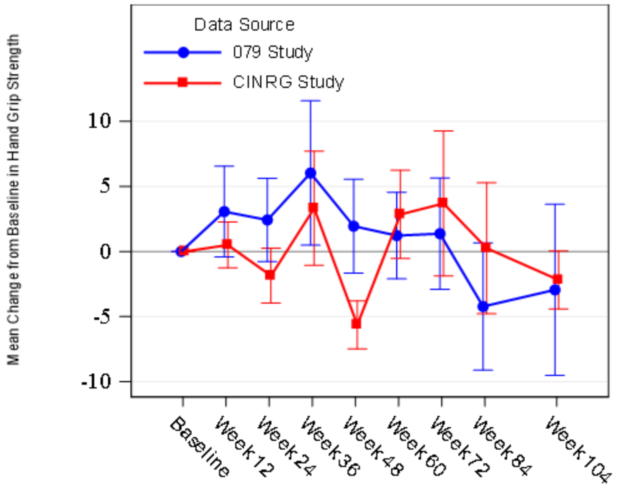

Group/n:									
**079 Study**	21	21	20	20	19	17	18	19	18
**CINRG Study**	36	10	11	13	20	11	7	6	21

Abbreviations: ppFVC = percent predicted forced vital capacity; CINRG = Cooperative International Neuromuscular Research Group; DMD = Duchenne muscular dystrophy; DNHS = DMD Natural History Study.

Supplementary Appendix S8 PK parameters following a single dose of pamrevlumab (*n*=12)

**T_{max} (hr), median (range)**	**C_{max} (µg/mL), mean (SD)**	**AUC_{336} (hr* µg/mL), mean (SD)**	**$t_{1/2}$ (day), mean (SD)**	**CL (mL/hr/kg), mean (SD)**	**V_{ss} (mL/kg), mean (SD)**
2.7 (1.8, 4.3)	969.7 (306.7)	143,459.5 (32,892.6)	9.2 (1.9)	0.17 (0.04)	52.0 (16.9)

Abbreviations: AUC = area under the curve; CL = clearance; PK = pharmacokinetic; SD = standard deviation; V_{ss} = apparent volume of distribution at steady state. PK parameters were calculated from the concentration versus time data from each patient by standard noncompartmental methods (Phoenix64®, WinNonlin®, Build 8.1, Certara, Princeton, NJ).

REFERENCES

[1] Mayhew AG, Coratti G, Mazzone ES, Klingels K, James M, Pane M, et al. Performance of Upper Limb module for Duchenne muscular dystrophy. Dev Med Child Neurol. 2020;62(5):633–9. doi:10.1111/dmcn.14361.

[2] Ricotti V, Selby V, Ridout D, Domingos J, Decostre V, Mayhew A, et al. Respiratory and upper limb function as outcome measures in ambulant and non-ambulant subjects with Duchenne muscular dystrophy: a prospective multicentre study. Neuromuscul Disord. 2019;29(4):261–8. doi:10.1016/j.nmd.2019.02.002.

[3] Meier T, Rummey C, Leinonen M, Spagnolo P, Mayer AH, Buyse GM, et al. Characterization of pulmonary function in 10–18-year-old patients with Duchenne muscular dystrophy. Neuromuscul Disord. 2017;27(4):307–14. doi:10.1016/j.nmd.2016.12.014.

[4] Pane M, Coratti G, Brogna C, Mazzone ES, Mayhew A, Fanelli L, et al. Upper limb function in Duchenne muscular dystrophy: 24-month longitudinal data. PLoS ONE. 2018;13(6):e0199223. doi:10.1371/journal.pone.0199223.

[5] Seferian AM, Moraux A, Annoussamy M, Canal A, Decostre V, Diabate O, et al. Upper limb strength and function changes during a one-year follow-up in non-ambulant patients with Duchenne muscular dystrophy: an observational multicenter trial. PLoS ONE. 2015;10(2):e0113999. doi:10.1371/journal.pone.0113999.
